# Retraction of: CCNA2 and KIF23 are molecular targets for the prognosis of adenoid cystic carcinoma

**DOI:** 10.18632/aging.206185

**Published:** 2024-12-31

**Authors:** Yongbin Di, Haolei Zhang, Bohao Zhang, Tianke Li, Dan Li

**Affiliations:** 1 Department of Stomatology, The First Hospital of Hebei Medical University, Shijiazhuang, Hebei 050030, P.R. China; 2 Department of Otolaryngology, Head and Neck Surgery, The First Hospital of Hebei Medical University, Shijiazhuang, Hebei 050030, P.R. China; 3 Department of Stomatology, The Fourth Hospital of Hebei Medical University, Shijiazhuang, Hebei 050011, P.R. China

**Keywords:** CCNA2, KIF23, prognosis, adenoid cystic carcinoma, bioinformatics

**This article has been retracted:** The Editors found inconsistencies in this paper after advance publication online ahead of its printing and contacted the authors for the clarification. The editorial concerns were not answered and include:

1. In the Abstract and Materials and Methods there is mention of Adenoid Cystic Carcinoma groups with gene overexpression or knockout. Based on that, one can conclude that there was genetic manipulation of the cell lines. Consistent with that conclusion, the final sentence in the Introduction states that, “The study validated the significant role of CCNA2 and KIF23 in adenoid cystic carcinoma by analyzing public datasets and further confirmed these findings through basic cell experiments.” At the same time, there is no mention of the cell lines or any gene modification in the Materials and Methods and Results.

2. Based on the above, readers expect to see validation experiments (Western blots) with the genetically modified cell lines. Instead, protein preparation from the tissues is described but there is no description of which genes were analyzed or modified.

Based on the absence of authors’ clarification, the Editorial decision was made to retract this paper. The authors were notified about the Editorial decision but they did not reply.

